# Structural Insights into Protein Regulation by Phosphorylation and Substrate Recognition of Protein Kinases/Phosphatases

**DOI:** 10.3390/life11090957

**Published:** 2021-09-13

**Authors:** Seung-Hyeon Seok

**Affiliations:** 1 College of Pharmacy, Jeju National University, Jeju 63243, Korea; sseok@jejunu.ac.kr; Tel.: +82-64-754-8193; 2 Jeju Research Institute of Pharmaceutical Sciences, Jeju National University, Jeju 63243, Korea; 3 Interdisciplinary Graduate Program in Advanced Convergence Technology & Science, Jeju National University, Jeju 63243, Korea

**Keywords:** protein phosphorylation, protein kinase, protein phosphatase, SLiMs, substrate recognition, structural regulation, protein structure

## Abstract

Protein phosphorylation is one of the most widely observed and important post-translational modification (PTM) processes. Protein phosphorylation is regulated by protein kinases, each of which covalently attaches a phosphate group to an amino acid side chain on a serine (Ser), threonine (Thr), or tyrosine (Tyr) residue of a protein, and by protein phosphatases, each of which, conversely, removes a phosphate group from a phosphoprotein. These reversible enzyme activities provide a regulatory mechanism by activating or deactivating many diverse functions of proteins in various cellular processes. In this review, their structures and substrate recognition are described and summarized, focusing on Ser/Thr protein kinases and protein Ser/Thr phosphatases, and the regulation of protein structures by phosphorylation. The studies reviewed here and the resulting information could contribute to further structural, biochemical, and combined studies on the mechanisms of protein phosphorylation and to drug discovery approaches targeting protein kinases or protein phosphatases.

## 1. Introduction

**Figure 1:**
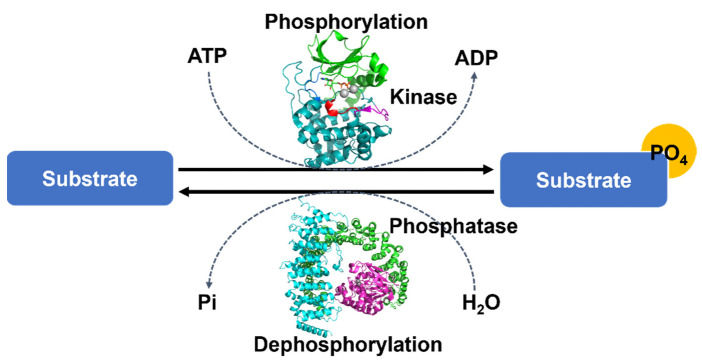
The overall mechanism of protein phosphorylation regulated by protein kinases and protein phosphatase. Each protein kinase covalently attaches a phosphate group from ATP to a protein substrate and each protein phosphatase removes a phosphate group from a phosphoprotein. These processes are reversible. Protein structures were drawn by the programs PyMOL (The PyMOL Molecular Graphics System, Version 2.4.1 Schrödinger, LLC., Cambridge, MA, USA).

Post-translational modification (PTM) is a key mechanism providing the functional diversity of proteins in cellular signaling and physiology and changing the functions or stability of proteins. Protein phosphorylation is one of the most widely observed and important PTM processes [1]. Protein phosphorylation is regulated by protein kinases, each of which covalently attaches a phosphate group to an amino acid side chain on serine (Ser), threonine (Thr), or tyrosine (Tyr), and by protein phosphatases, each of which, conversely, removes a phosphate group from a phosphoprotein (Figure 1). These reversible enzyme activities provide a regulatory mechanism, altering the changing diverse functions and stability of proteins in cellular processes and the diverse physiological functions related to musculoskeletal regulation, neurologic mechanisms and behavior, immune response, endocrine action, and so forth [2,3].

More than two-thirds of over 20,000 proteins from human proteomes have been reported to be phosphorylated, and over 200,000 unique phosphorylation sites have been detected [4]. Reflecting the importance and abundance of protein phosphorylation, the human genome encodes more than 500 protein kinases and ~180 protein phosphatases [5]. Although the activities of protein kinases and protein phosphatases are counteracting, the ratio of the number of kinase to phosphatase species is highly unbalanced. Due to the dynamic assembly of phosphatase catalytic subunits into diverse holoenzymes that target substrates, the unbalance is, however, resolved [6]. Most phosphorylation sites in proteins are localized on their disordered or dynamic regions [7,8,9], and the reversible attachment or detachment of phosphate groups in these regions causes changes in their molecular structures to induce protein conformational shifts, interference of protein–protein or protein–nucleic acid interactions, or disorder-to-order or order-to-disorder transitions in proteins [10,11]. The structural flexibility of proteins makes it challenging to study the structural basis of kinase and phosphatase activities and the reversible phosphorylation/dephosphorylation mechanisms.

In this review, the structures and substrate recognition of protein kinases/phosphatases are described and summarized, focusing on Ser/Thr protein kinases and protein Ser/Thr phosphatases, and the structural regulation of proteins by their phosphorylation. The studies reviewed here and the resulting information could contribute to further structural, biochemical, and combined studies on the mechanisms of protein phosphorylation and to drug discovery approaches targeting protein kinases or protein phosphatases.

## 2. Ser/Thr Protein Kinase Folding

Protein kinases each catalyze the enzymatic reaction of removing a phosphate group from ATP and covalently attaching it to one of three hydroxyl-containing amino acids, Ser, Thr, and Tyr, in eukaryotes. Most phosphorylation events occur on Ser (86%), followed by Thr (12%) and Tyr (2%) [12]. Phosphorylation events on other amino acids, including histidine and arginine, are infrequent in eukaryotes and less stable [13,14]. 

Based on the phosphorylation of the residues in the target proteins, most human protein kinases are classified into two families: Ser/Thr protein kinases, which act on Ser/Thr residues, and tyrosine protein kinases, which act on Tyr residues [4,15]. Reflecting the number of phosphorylation events on amino acids, the human genome includes more than 400 Ser/Thr protein kinases and ~100 tyrosine protein kinases [16,17]. In addition, some minor protein kinases act on histidine (His), aspartic acid (Asp), or glutamic acid (Glu) residues [13,14].

### 2.1. General Folding of Ser/Thr Protein Kinase

Most kinases share a catalytic domain (protein kinase domain) that is a highly conserved homologous region of ~290 residues. Each kinase contains a small N-terminal lobe, predominantly composed of five β-sheets and an α-helix (called the C-helix), and a larger C-terminal lobe, predominantly composed of six helices, an activation loop, and a catalytic loop [18]. The activation loop contains the Asp-Phe-Gly (DFG) motif, which supports the binding of Mg^2+^ ions to ATP by chelating them in the active conformation of each protein kinase. The catalytic loop contains the His-Arg-Asp (HRD) motif, which interacts directly with the hydroxyl group of a Ser, Thr, or Tyr residue and transfers the ATP γ-phosphate to the protein substrate [19]. Upon binding of ATP and the protein substrate, the two lobes rotate the terminal phosphate group of the ATP and the target residue of the substrate into their correct positions for the catalytic reaction [20,21].

### 2.2. Protein Kinase A

**Figure 2:**
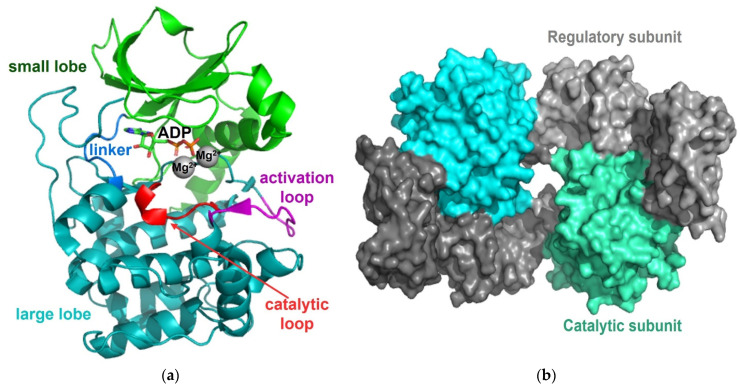
Structure of PKA. (**a**) Structure of the catalytic subunit of PKA (PDB code: 3TNQ) consisting of a small lobe (green) and a large lobe (teal), both of which are connected with a linker (blue). ADP and two magnesium ions (grey) are in the active site neighboring the activation loop (magenta) and the catalytic loop (red). (**b**) Overall structure of the PKA holoenzyme (PDB code: 3TNP) consisting of two regulatory subunits (grey and dark grey) and two catalytic subunits (cyan and green cyan). Upon binding of cAMP to the regulatory subunits, two active catalytic subunits are released.

Protein Kinase A (PKA, cAMP-dependent protein kinase) is one of the most well-known and researched protein kinases and plays key roles in numerous biological processes, including metabolic regulation, inflammation, hormone secretion, and various types of signal transduction [22,23]. Reflecting the diverse roles of PKA, thousands of substrates for PKA have been screened, discovered, and reported [24,25,26]. Similar to other protein kinases, the highly dynamic catalytic subunit of PKA also shares the conserved N-terminal and C-terminal lobes linked with a hinge region and contains the conserved catalytic loop, located in the interface of the domains, and the conserved activation loop, located between the large C-terminal lobe and the C-helix in the N-terminal lobe [27,28,29,30]. The substrate binding site is flanked by N- and C-terminal lobes in an extended conformation that may be involved in regulation (Figure 2a) [28,31,32]. The regulatory subunit contains a catalytic subunit-binding domain and two cAMP-binding domains, and it forms a homodimer in an anti-parallel orientation (Figure 2b). In the inactive conformation, the PKA holoenzyme is in the heterotetrametric conformation, composed of two regulatory subunits and two catalytic subunits in closed conformation [33]. Four cAMP molecules can bind to a PKA holoenzyme (one molecule of cAMP binds to each of the two cAMP-binding sites on a regulatory subunit), causing an allosteric conformational change in the regulatory subunits and the catalytic subunits and the subsequent release of two active catalytic subunits in open conformation [32,34].

### 2.3. Cyclin-Dependent Kinase 2

**Figure 3:**
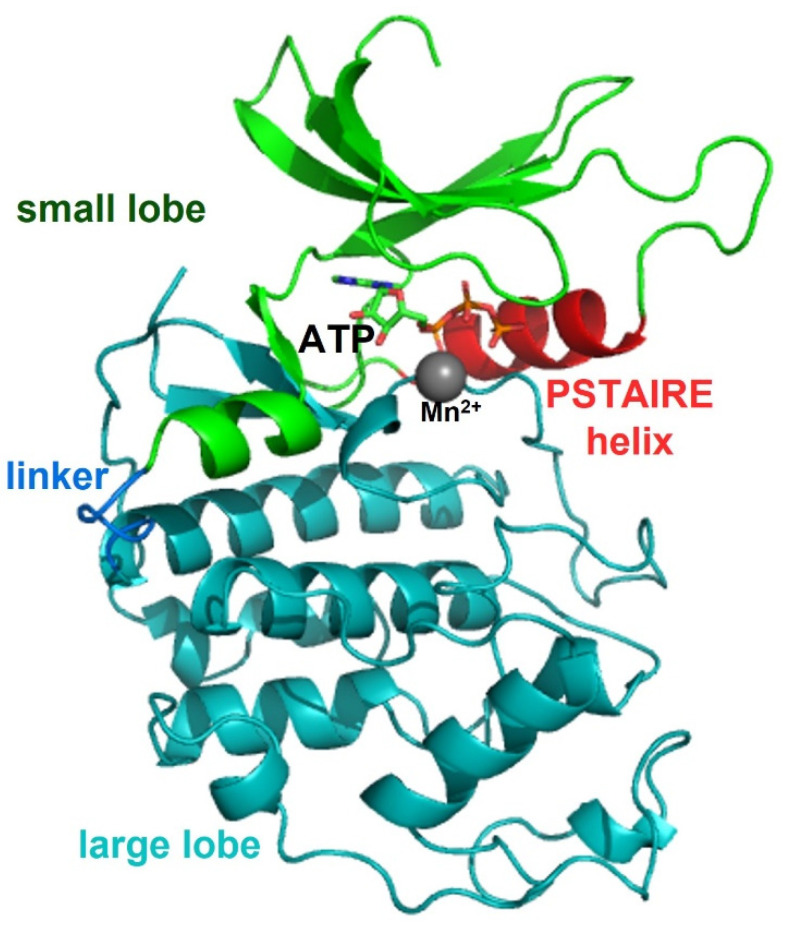
Structure of the catalytic subunit of CDK2 (PDB code: 1JST). The catalytic subunit of CDK2 is also structured in conserved N-terminal (green) and C-terminal (teal) lobes, both of which are connected with a linker (blue). The conserved PSTAIRE helix (red) located in the N-terminal lobe is a major interaction region with cyclin via hydrophobic interactions with another helix in cyclin.

Cyclin-dependent kinase 2 (CDK2) is a member of the cyclin-dependent kinase family of Ser/Thr protein kinases and one of the key enzymes in the regulation of the cell cycle, phosphorylating diverse proteins involved in multiple cellular processes [35,36]. The CKD2 activity is regulated by association with an activating partner protein in the cyclin family and through phosphorylates of conserved Thr residue in this kinase [37]. CDK2 is also structured in a conserved N-terminal lobe and a C-terminal lobe. In the inactive state, the activation loop in the C-terminal lobe causes steric hindrance in the active site and the protein substrate-binding site, leading to an incorrect orientation of the phosphate atoms in ATP for the kinase reaction. When cyclin binds to the active site located between the N- and C-terminal lobes of the CDK2, cyclin also interacts with both the N- and C-terminal lobes. The conserved PSTAIRE helix located in the N-terminal lobe is a major interaction region with cyclin via hydrophobic interactions with another helix in cyclin. In other words, cyclin causes conformational changes in CDK2 so that the PSTAIRE helix rotates inward and moves closer to the C-terminal lobe of CDK2 (Figure 3). This conformational change in CDK2 also aids the relocation of the residues in the activation loop and the interaction of related residues with the phosphate groups of ATP, allowing the conserved Thr residue to be exposed and phosphorylated as the activation loop is displaced from the active site [38,39].

## 3. Protein Ser/Thr Phosphatase Folding

The extremely high stability of phosphorylated residues means that dephosphorylation by protein phosphatases is essential for the regulation of the dynamic and reversible states of proteins. As a counterreaction with protein kinases, each protein phosphatase removes a phosphate group from the phosphorylated amino acid residue of its substrate protein [40].

Most protein phosphatases can also be classified into two families, protein Ser/Thr phosphatases and protein Tyr phosphatases, depending on the dephosphorylation of the phosphorylated residues in their target proteins or substrates. In this review, some minor dephosphorylation events that occur on the phosphohistidine will be excluded and members of the protein Ser/Thr phosphatases will be described. As described above, the human genome contains more than 500 protein kinases, whereas it contains around 180 protein phosphatases [5]. Furthermore, the number of protein Ser/Thr phosphatases encoded in the human genome (~50) is 10 times lower than the number of Ser/Thr protein kinases encoded in the human genome [5,17]. This gap between protein Ser/Thr phosphatases and Ser/Thr protein kinases could be explained by the dynamic assembly and combinatorial conformation of holoenzymes with shared catalytic subunits of protein Ser/Thr phosphatases and the diverse regulatory subunits that target distinct proteins [41,42].

The protein Ser/Thr phosphatases can be divided into two families, phosphoprotein phosphatases and metal-dependent protein phosphatases, based on their sequence homology and catalytic metal dependence [43]. The phosphoprotein phosphatase family, genetically encoding approximately 15 human proteins and Zn/Fe-dependent enzymes, includes protein phosphatase 1 (PP1), PP2A, PP2B (calcineurin), PP4, PP5, PP6, and PP7 [4,44]. Most phosphoprotein phosphatases share a conserved 30 kD catalytic domain containing highly conserved sequences, GDxHG, GDxVDRG, and GNHE.

### 3.1. Protein Phosphatase 1

**Figure 4:**
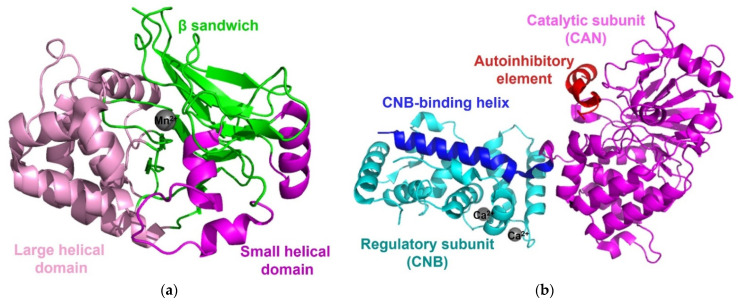
Structure of PP1 and calcineurin. (**a**) The structure of PP1 (PDB code: 6OBQ) contains a β-sandwich (green), flanked by a large helical domain (pink) and a small helical domain (magenta), and two manganese ions (grey). (**b**) The overall structure of calcineurin (PDB code: 4OR9) consists of a catalytic subunit (CNA, magenta), a regulatory subunit (CNB, cyan), and two calcium ions (grey). The CNB-binding helix (blue) is extended to CNB and the autoinhibitory element (red) of CNA forms an α-helix and then binds to surface residues on the phosphatase domain of the catalytic center.

PP1, the most widely expressed protein Ser/Thr phosphatase that is responsible for more than 50% of all dephosphorylation reactions in humans, plays a key role in the regulation of a wide range of cellular processes regulated. PP1 is one of the simplest phosphatases and consists of only a highly conserved catalytic subunit, which is associated with at least one of 200 known regulatory proteins. The catalytic domain of PP1 comprises a central β-sandwich formed by two mixed β-sheets surrounded by two α-helical domains on both sides [45,46]. Two metal ions (Mn^2+^ or Fe^2+^), located in the active site of a central β-sandwich, are coordinated with six highly conserved residues, three histidines, two aspartic acids, and one asparagine. The binding and activation of a water molecule by two metal ions initiates a nucleophilic attack on the phosphorous atom (Figure 4a) [45,46].

### 3.2. Calcineurin/Protein Phosphatase 2B

Calcineurin (also known as Protein Phosphatase 2B, PP2B) regulates diverse calcium-dependent biological processes, such as neurodevelopment and memory, cardiac hypertrophy, signal transduction, muscle development, and the immune response [47]. Calcineurin consists of a ~60 kD calmodulin-binding catalytic subunit (calcineurin A or CNA) and a ~20 kD regulatory subunit (calcineurin B or CNB). The CNA subunit is highly conserved and similar to the catalytic subunit of PP1, with an identical pattern of metal ion coordination [48], and it contains an N-terminal phosphatase domain, a CNB-binding helical domain, a Ca^2+^-calmodulin (Ca^2+^-CaM) binding motif, and an autoinhibitory element. Calcineurin alone is inactive, and its phosphatase activity is activated upon interaction with Ca^2+^-CaM. Because the disordered autoinhibitory element of CNA forms an α-helix and then binds to surface residues on the phosphatase domain through a combination of hydrogen bonds and van der Waals interactions, access to the catalytic center can be blocked (Figure 4b) [43,48]. Although the CaM-dependent activation of calcineurin is clear, the structural information about its CaM-dependent activation mechanism remains to be clarified, because little structural information about the calcineurin-CaM complex has been reported to date. All calcineurin structures have been determined in the absence of CaM or in complex with small fragments of CaM [49,50,51,52,53,54,55]. However, combining approaches with diverse structural, biochemical, and biophysical analyses has revealed how calcineurin is activated by Ca^2+^-CaM. Upon binding of Ca^2+^-CaM to calcineurin, an autoinhibitory element becomes ordered, resulting in a stable helical structure. As a result, the displacement of the disordered autoinhibitory element from the catalytic center causes calcineurin to be activated [56,57].

### 3.3. Protein Phosphatase 2A

**Figure 5:**
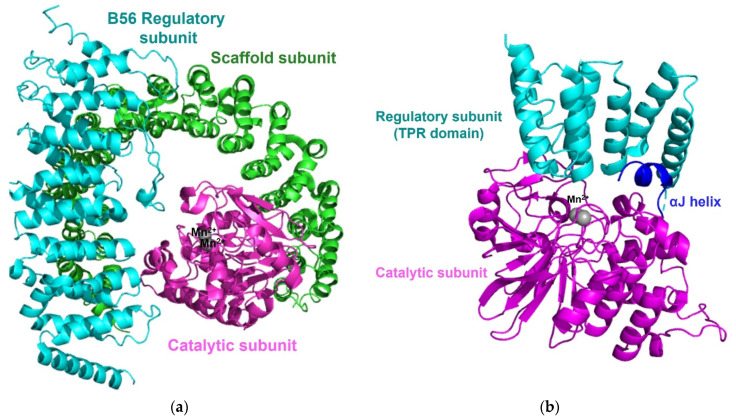
Overall structure of the PP2A holoenzyme and PP5. (**a**) The overall structure of the PP2A holoenzyme harboring a B56 regulatory subunits (PDB code: 2NYL) consists of a scaffold subunit (green), a catalytic subunit (magenta) with two manganese ions (grey), and a B56 regulatory subunit (cyan). (**b**) The structure of PP5 (PDB code: 1WAO) contains a catalytic domain (magenta), a regulatory domain (TPR domain, cyan), and two manganese ions (grey). The interaction between the C-terminal αJ-helix (blue) and the N-terminal TPR domain suppresses the phosphatases activity of free PP5 and maintains an autoinhibited conformation.

Protein phosphatase 2A (PP2A), one of the most abundant enzymes in humans, represents up to 1% of the total cellular protein in several tissues. PP2A regulates cellular processes, normal physiologies, and numerous signaling pathways [58,59]. Cellular PP2A enzymes exist in either a heterodimeric core enzyme or a heterotrimeric holoenzyme. The PP2A heterodimeric core enzyme comprises a 65 kD scaffold subunit (also known as the A, PR65A, or PPP2R1 subunit), containing 15 tandem HEAT repeats and forms a horseshoe-shaped structure, and a 35kD catalytic subunit (PP2AC and PPP2C subunit), which recognizes the conserved ridge of HEAT repeats 11-15 for association [60,61]. The PP2A core enzyme forms an active heterotrimeric holoenzyme by assembly with one of four regulatory subunits: B (B55, PR55, or PPP2R2), B′ (B56, PR61, or PPPP2R5, B′′ (PR48/PR70/PR130 or PPPP2R3), and B′′′ (Striatins or PR93/PR110) (Figure 5a) [43,58]. While the sequences of the scaffold subunit and the catalytic subunit show high conservation among all eukaryotes, the regulatory subunits are more heterogeneous and play key roles in controlling the specific activity and the substrate selectivity of different holoenzymes. The structure of the PP2A holoenzyme containing the B’ subunit shows that the B’ subunit contains eight HEAT-like repeats and interacts with both the scaffold subunit and the catalytic subunit [62]. In the structure of the PP2A holoenzyme harboring the regulatory B subunit, the B subunit containing seven WD40 repeats participates in few interactions with the catalytic subunit, unlike the PP2A holoenzyme containing the B’ subunit [63]. In each PP2A holoenzyme structure, the potential substrate-binding site is on the top surface of the regulatory subunit and located close to the active site of the catalytic subunit to target substrate phosphoproteins [61,62,63].

### 3.4. Other Protein Ser/Thr Phosphatases

Each protein phosphatase 4 (PP4), 5 (PP5), and 6 (PP6) also has a conserved catalytic core domain, which resembles the domain in PP1 or PP2A. The catalytic subunit of PP4 associates with its own regulatory subunits R1 or R2 [64] and the catalytic subunit of PP6 forms a heterotrimeric holoenzyme with one of three Sit4-associated protein (SAP) domain-containing subunits (PPP6R1-3 or SAPS 1-3) and one of three ankyrin repeat domain subunits (ANR28, ANR44, and ANR52) that serves as the regulatory subunit [65,66]. Unlike most phosphoprotein phosphatases, PP5 is encoded by a single gene. PP5 contains the tetratricopeptide repeat (TPR) domain, a regulatory domain, at the N-terminus, and a catalytic domain, containing an αJ-helix, at the C-terminus. The interaction between the C-terminal αJ-helix and the N-terminal TPR domain suppresses the phosphatase activity of free PP5 and maintains an autoinhibited conformation [67].

### 3.5. Metal-Dependent Protein Phosphatases

The metal-dependent protein phosphatase (PPM) family, genetically encoded approximately 16 human proteins and Mn^2+^/Mg^2+^-dependent enzymes, includes PP2C and pyruvate dehydrogenase phosphatase [4,44]. PP2C plays a key role in the regulation of stress signaling and other cellular signaling [68]. The conserved catalytic core domain of human PP2C shows similar domain folding to other phosphoprotein phosphatases each containing a central β-sandwich, flanked by a pair of α-helices, and coordinating the two metal ions with amino acids and water molecules.

## 4. Substrate Recognition with Short Linear Motifs (SLiMs)

Since more than 500 protein kinases and ~180 protein phosphatases participate in the coordination of more than two-thirds of over 20,000 proteins from human proteomes, each protein kinase or protein phosphatase simultaneously has multiple substrates and diverse functions [4,5]. As specificity for the substrate recognition of protein kinases and protein phosphatases is critical, it is necessary to understand the substrate recognition mechanism of these enzymes. Most protein kinases or protein phosphatases exhibit their specificity through the recognition of consensus residues around the phosphorylation sites [6,15,69]. The short motifs harboring consensus residues are called short linear-interaction motifs (SLiMs), each consisting of 4–10 amino acids, and mediating interactions with proteins of low affinity [70,71]. Most SLiMs are in disordered or less ordered regions, allowing flexibility for interactions with protein kinases or protein phosphatases [15,69]. 

Consensus sequences of SLiMs for several Ser/Thr protein kinases and protein Ser/Thr phosphatases are summarized in Table 1. Consensus sequences of SLiMs for protein kinases are characterized and suggested from structural studies on protein kinases in complex with their protein substrates or peptide substrates. In addition to structural approaches, mass spectrometry-based proteomics, phage display, peptide arrays, and computational approaches have been used as major tools for identifying the residues required for optimal substrate phosphorylation/dephosphorylation [72,73,74,75,76]. 

Whereas SLiMs for many kinases have been well-characterized and studied, there is a lack of knowledge regarding substrate recognition and SLiMs for protein phosphatase complexes. Despite shared structural features in the catalytic subunits among phosphoprotein phosphatases, most catalytic subunits of phosphoprotein phosphatases do not exist as free conformations in cells but are in association with regulatory subunits and other proteins. Due to the lack of structural knowledge regarding the catalytic subunits of phosphoprotein phosphatases, studies on substrate recognition and SLiMs for these enzymes are relatively difficult and only a few SLiMs of phosphoprotein phosphatases have been identified.

**Figure 6:**
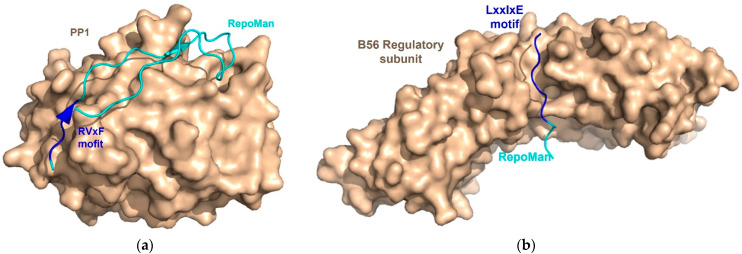
Structural insights into the binding of SLiMs to phosphatases. (**a**) Structure of PP1 in complex with RepoMan (PDB code: 5IOH). The consensus sequence, RVxF (blue), is positioned on the surface of PP1 (tints). (**b**) Complex structure (PDB code: 5SW9) of the B56 regulatory subunit of PP2A (tints) with RepoMan (cyan) containing the LxxIxE motif (blue), a SLiM, for the B56 regulatory subunit.

One of the most well-known and the first discovered SLiMs for phosphoprotein phosphatases is the RVxF motif that binds to PP1, reported in the complex structure of the catalytic subunit of PP1 and 13mer of the G_M_-peptide. It was found that the side chains of the RVxF motif interact with a hydrophobic pocket on the PP1 surface (Figure 6a) [86]. 

Unlike PP1, PP2A forms a heterotrimeric holoenzyme complexed with a heterodimeric core enzyme (scaffold subunit A and catalytic subunit C) and one of the various regulatory subunits that controls specific substrate recognition. Due to this complexity of the PP2A holoenzyme, only one SLiM for the B56 regulatory subunit has been clearly reported and identified. A structural study of the PP2A harboring B56 regulatory subunit with RepoMan reported that the LxxIxE sequence binds to the concave surface of HEAT repeats 3 and 4 in the B56 regulatory subunit [87]. Recently, multiple studies using phage display, peptide arrays, and computational approaches have validated this and suggest a LxxIxE motif as the preferred docking site for the B56 regulatory subunit (Figure 6b) [74,76,91,92].

In the case of calcineurin, the PxIxIT motif has been suggested and validated as a SLiM following multiple structural analyses [88,89,90,93]. Although the PxIxIT motif is conserved and identified as a preferred SLiM for substrate recognition, additional binding elements from the substrate are required for the specific activity of calcineurin. Some structural studies on calcineurin complexed with a viral protein inhibitor, FK506, or cyclosporin report an additional LxVP motif as a SLiM for calcineurin that is critical for substrate recognition [48,52,88].

## 5. Structural Regulation by Phosphorylation 

Phosphorylation is a rapid and reversible kinetic enzymatic reaction that regulates protein activity and cellular processes via the attachment and detachment of a dianionic phosphate group on the residue of a protein, providing a change in the structural properties of proteins and regulation of protein–protein interactions. Phosphoryl groups of phosphorylated residues can form extensive hydrogen bonds and salt bridges between the phosphate oxygens and neighboring residues that affect the stability, dynamics, and structural properties of proteins [94]. 

Because most phosphorylation or dephosphorylation sites of Ser/Thr are localized on disordered or less-ordered regions, disorder-to-order or order-to-disorder transitions are one of the key regulators of protein functions [7,8,9]. Centromere protein T (CENP-T) has a long N-terminal disordered region that folds upon phosphorylation on Thr72, located at the center of the disordered region. This disorder–order transition enhances the interaction with the Spc24/Spc25 subunits of Ndc80 [95,96]. In addition, protein phosphorylation also regulates the folding of intrinsically disordered proteins, which play important roles in cell signaling, transcription, translation, and cellular processes. In the case of eukaryotic translation initiation factor 4E-binding protein 2 (4E-BP2), whereas the phosphorylation of 4E-BP2 induces protein fold stabilization [97], the eIF4E:4E-BP2 interaction was found to be weakened by phosphorylation-induced disorder–to-order transition of 4E-BP2 [98]. In addition, localized disorder-to-order transition on phosphorylated RNA binding proteins has been reported [99].

Protein phosphorylation can also impact protein structure at local as well as global levels. Phenylalanine hydroxylase (PAH), which catalyzes the hydroxylation of L-phenylalanine to L-tyrosine, is regulated by phosphorylation by PKA; the structural stability of PAH is enhanced by phosphorylation-induced localized structural changes [100]. In the case of bromodomain-containing protein 4 (BRD4), an epigenetic regulator and a transcription cofactor, it is activated by protein kinase CK2 (casein kinase 2; CK2) and is inactivated by PP2A. When BRD4 is inactivated by dephosphorylation, intramolecular contact is formed between conserved bromodomain 2 (BD2) and the N-terminal cluster of CK2 phosphorylation sites (NPS), containing multiple serine residues. Upon CK2-mediated phosphorylation of NPS, its intramolecular contact is switched to a downstream basic residue-enriched interaction domain (BID) and generates conformational changes for the recruitment of sequence-specific transcription factors [101,102].

## 6. Conclusions

The PhosphoSitePlus database suggests that there are more than 200,000 phosphorylation sites on Ser and Thr residues [103,104]. This is consistent with more than 400 Ser/Thr protein kinases encoded in the human genome. Despite its abundance and importance, there remains much to be learned about protein phosphorylation. Questions about the specificity and regulation of Ser/Thr phosphatases are more difficult to answer. The elucidation of regulatory mechanisms and substrate recognition processes for protein Ser/Thr phosphatases is also complicated because of the existence of multiple regulatory subunits and other interacting proteins and due to the lack of determined structures of the complexes of catalytic and regulatory subunits. To overcome these difficulties, several approaches, such as advanced structural analysis, mass spectrometry-based proteomics, phage display, peptide arrays, and computational approaches, have been utilized and combined [72,73,74,75,76].

This review focuses on the structures and substrate recognition of Ser/Thr protein kinases and protein Ser/Thr phosphatases, and protein structural regulation by phosphorylation has also been briefly summarized and described. Although most kinases or phosphatases share conserved structural features in their respective catalytic subunit, these proteins regulate the specificity of enzymatic activity through association with regulatory subunits and substrate recognition through binding to SLiMs. The resultant reversible attachment and detachment of phosphates causes changes in molecular structure that induce conformational shifts, interference with protein–protein interactions, or disorder-to-order or order-to-disorder transitions in proteins [10,11]. The use of the structural insights provided in this review is expected to support further structural, biochemical, and multidisciplinary studies to reveal the mechanisms behind protein phosphorylation and provide knowledge for drug discovery efforts targeting protein phosphorylation.

## Figures and Tables

**Table 1:** Consensus sequences of SLiMs recognized by Ser/Thr protein kinases or protein Ser/Thr phosphatases.

Name	Consensus Sequence ^1,2,3^	References
**Ser/Thr** **protein kinases**		
Cyclic AMP-dependent kinase (PKA)	R-R/K-S/T-ϕ	[77]
Protein kinase B (PKB, Akt)	R-x-R-x-x-S/T-ϕ	[15]
Cyclic GMP-dependent protein kinase (PKG)	R/K-R/K-R/K-x-S/T-x	[77]
Protein kinase C (PKC)	x-S/T-x-R/K	[78]
Cyclin-dependent kinases (CDKs)	S/T-P-x-K/R	[79]
Casein kinase 1 (CK1)	D/E-D/E-D/E-x-x-S/T-ϕ pS/pT-x-x-S/T-ϕ	[80]
Casein kinase 2 (CK2)	S/T-D/E-x-D/E	[81]
Calcium/Calmodulin-dependent Protein Kinase II (CaM II)	R-x-x-S/T-x	[77]
AMP-activated protein kinase (AMPK)	ϕ-x-R-x-x-S-x-x-x-I/L	[82]
Phosphorylase kinase (PhK)	R-x-x-S/T-x-ϕ-R	[15]
Mitogen-activated protein kinases (MAPKs)	P/ϕ-x-S/T-P	[83]
NimA-related kinase (NEK)	ϕ-x-x-S/T	[82]
Polo-kike kinase 1 (Plk)	D/E/N-x-S/T-ϕ	[84]
**Protein Ser/Thr** **phosphatase**		
Protein phosphatase 1 (PP1)	R-V-x-F	[85,86]
Protein phosphatase 2A-B56 (PP2A-B56)	L-x-x-I-x-E	[74,87]
Calcineurin (PP2B)	P-x-I-x-I-T LxVP	[88,89,90]

^1^ x is any amino acid. ^2^ ϕ is a hydrophobic residue. ^3^ Phosphorylated residues are underlined.
